# The Value of Circulating Tumor Cells in the Prognosis and Treatment of Pancreatic Cancer

**DOI:** 10.3389/fonc.2022.933645

**Published:** 2022-07-04

**Authors:** Kai Luo, Xiangkun Wang, Xudong Zhang, Zhongyuan Liu, Shuai Huang, Renfeng Li

**Affiliations:** Department of Hepatobiliary Surgery, The First Affiliated Hospital of Zhengzhou University, Zhengzhou, China

**Keywords:** circulating tumor cell, pancreatic carcinoma, EMT, prognosis, circulating tumor cell (CTCs)

## Abstract

In the past few decades, tumor diagnosis and treatment theory have developed in a variety of directions. The number of people dying from pancreatic cancer increases while the mortality rate of other common tumors decreases. Traditional imaging methods show the boundaries of pancreatic tumor, but they are not sufficient to judge early micrometastasis. Although carcinoembryonic antigen (CEA) and carbohydrate antigen19-9 (CA19-9) have the obvious advantages of simplicity and minimal invasiveness, these biomarkers obviously lack sensitivity and specificity. Circulating tumor cells (CTCs) have attracted attention as a non-invasive, dynamic, and real-time liquid biopsy technique for analyzing tumor characteristics. With the continuous development of new CTCs enrichment technologies, substantial progress has been made in the basic research of CTCs clinical application prospects. In many metastatic cancers, CTCs have been studied as an independent prognostic factor. This article reviews the research progress of CTCs in the treatment and prognosis of pancreatic cancer.

## Introduction

As one of the most malignant abdominal tumors, pancreatic adenocarcinoma (PDAC) is the fourth leading cause of tumor-related death in Western countries (1). A predictive study of the burden of cancer in 28 European countries using a time-linear Poisson regression model showed that pancreatic cancer will surpass breast cancer as the third leading cause of cancer-related deaths by 2025 (2, 3). By 2030, it will be the second leading cause of cancer-related deaths in the United States (3). In the past few decades, despite the great progress made in adjuvant therapy, neoadjuvant therapy, and chemotherapy, the prognosis of pancreatic cancer is still very poor. Although multidisciplinary precision treatment has been applied, and fine perioperative management has been implemented, the median survival time is less than 3–6 months, and less than 9% of diagnosed patients survive beyond 5 years (4, 5). The main reason for these dismal results is the difficulty in early diagnosis. The highly malignant nature of PDAC manifested as highly invasive early metastasis and unremarkable symptoms before the tumors evolve from the early stage to the late stage, which means that surgical treatment is not feasible. Still, we have no approach to stratify the risks for tumor metastasis, which are key indicators to guide neoadjuvant and adjuvant therapies, despite detecting the tumor in the early stage. The pathogenesis and etiology of PDAC are complicated and multifactorial, consisting of genetic and environmental factors. According to the United States Preventive Services Task Force (USPSTF) epidemiological study of pancreatic cancer, type 2 diabetes, obesity, advanced age, history of smoking, and chronic pancreatitis are predominant risk factors of PDAC (6).

Computed tomography (CT) is still the most effective means for clinical diagnosis and staging of PDAC and is a common method to monitor treatment response. CT and magnetic resonance imaging (MRI) display the anatomical boundaries of the tumor lesion, but they are inefficient to accurately assess the presence of tumor metastasis especially very small metastases in the early stage. Positron emission tomography (PET) is also incapable to detect micrometastases of PDAC because of the resolution ratio limitation that ranged from 4 to 10 mm (7). The ability to detect stage T1 pancreatic cancer with a tumor diameter of less than 2 cm can be used as a measure of various imaging techniques to diagnose early-stage pancreatic cancer (**Table 1**). Based on the comparison, it is clear that PET-CT is the imaging method with the highest detection rate for early-stage pancreatic cancer. PET-CT is useful as a whole-body examination in assessing the M-stage of tumors and has unique advantages in detecting distant metastases. Although PET-CT is more sensitive in detecting early pancreatic cancer, it is less specific and may give false-negative results for active chronic pancreatitis, plasmacytic cystadenoma, and enlarged lymph nodes in the head of the pancreas. Also, PET-CT is imprecise in its anatomical localization. Additionally, such imaging requires specialized and expensive equipment. Histological and cytological analysis is still the primary method to get an accurate diagnosis. Ultrasound-guided fine needle aspiration (FNA) is the most commonly used tissue acquisition method. Although the sensitivity of EUS seems to be the highest, this comes at the cost of invasive pathology (EUS-FNA). Unfortunately, the methods require superb expertise and there is a corresponding risk of complications. If the puncture extracts the matrix of the tumor tissue instead of cancer cells, it means a failure of diagnosis, and multiple fine needle aspirations may promote tumor proliferation.

**Table 1:** The ability of various imaging techniques to detect early-stage pancreatic carcinoma with a tumor diameter of less than 2 cm.

	Sensitivity	Ref
US	58%–78%	(8–10)
EUS	80%–100%	(8–17)
ERCP	84%–93%	(9, 18)
Dynamic enhanced CT	50%–77%	(11–17)
MRI	80%–87%	(19–21)
PET-CT	81%–100%	(22–25)

US, ultrasound; EUS, endoscopic ultrasonography; ERCP, endoscopic retrograde cholangiopancreatography; CT, computed tomography; MRI, magnetic resonance imaging; PET-CT, positron emission tomography-computed tomography.

The measurements of CA19-9 and CEA involve a few available blood-derived biomarkers. In particular, CA19-9 is the only serum biomarker approved by the United States Food and Drug Administration. Although blood-based tumor biomarkers have significant advantages (they are cheap, simple, and minimally invasive), these common tumor biomarkers have apparent limitations (they lack sensitivity and specificity); thus, they are not recommended for use in the early screening of pancreatic cancer. To date, the best marker, CA19-9, a sialylated Lewis antigen derived from the mucin 1 (MUC1) protein, has an overall sensitivity of 80% and a specificity of 82%. However, 5%–10% of the Caucasian population are incapable of synthesizing the CA19-9 epitope because of their Lewis a- and b‐genotype blood type (26). In addition, when we detect the blood of patients with benign diseases such as cholangitis, obstructive jaundice, and cirrhosis, CA19-9 is also elevated, thus increasing the difficulty of discrimination (27). On the other hand, CA19-9 does not increase in 35% of the patients diagnosed with resectable pancreatic cancer (28). However, for pancreatic cancers less than 1 cm in diameter and confined to the ductal epithelium, the 5-year survival rate after surgical resection can reach 100% (29). Therefore, we urgently need a reliable and all-purpose method to synthesize early detection of PDAC, real-time monitoring of radiotherapy and chemotherapy response, and prediction of the risk of tumor recurrence or metastasis.

Circulating tumor cells (CTCs) are tumor cells that have the capability to enter the blood circulating system. This cellular population circulates through peripancreatic vessels and their capillaries, along with tumor-induced neovessels, ultimately leading to metastasis progression in multiple organs (30). Research has shown that CTCs can enter the blood circulation in two ways: released from the tumor surface through shedding passively or by an active epithelial-to-mesenchymal transition (EMT) mechanism. Many tumor cells shed at the early stage of tumor formation through the first mechanism (31, 32). In the course of tumor development, some epithelial cells experience cellular changes in EMT. This change enhances their capacity of leaving the site of the primary tumor by increasing its mobility (33). CTCs analysis is beneficial in that its immunophenotypic and molecular genetic features may accurately represent the primary tumor serving as a “liquid biopsy” for metastatic tumors. At present, the two main assay methods widely used for CTCs detection are an immunomagnetic technique led by the CellSearch system and real-time quantitative PCR (qRT-PCR). A large number of derivative measurement technologies based on them are gradually being developed.

In recent years, with the continuous development of new CTCs enrichment technologies, substantial progress has been made in basic research on the prospects of CTCs clinical applications. For example, in many metastatic cancers, CTCs have been studied as an independent prognostic factor to predict patients’ progression-free survival (PFS) and overall survival (OS) (34–38). We found two studies that directly compared the diagnostic efficacy of CTCs and CA19-9. A prospective study in 2022 included blood samples from 82 patients with PDAC. This study aimed to compare the diagnostic efficacy of CTCs and CA19-9 in PDAC (39). In view of the false-negative results of CA19-9 in some Lewis antigen-negative populations, the researchers divided patients into a low-expression group (CA19-9 < 37 U/ml) and a high-expression group (CA19-9 ≥ 37 U/ml). In the low-expression group, 18 PDAC patients were misdiagnosed as negative by CA19-9, while CTCs correctly diagnosed 14 of the 18 misdiagnosed patients while maintaining high specificity. Zhang et al. obtained the patient’s portal vein blood to detect CTCs to evaluate their diagnostic and prognostic value for pancreatic cancer. The AUC demonstrated the diagnostic efficacy of different indicators: POV CTCs+CA19-9 (AUROC = 0.987), POV CTCs (AUROC = 0.942), and CA19-9 (AUROC = 0.806) (40). Their findings also suggested that the combination of CTCs and CA19-9 has a higher diagnostic power than individual indicators. In the neoadjuvant setting, the detection of CTCs is also an independent prognostic risk factor (41). In particular, strong evidence from epidemiological studies for CTCs as independent and stable prognostic factors has been published for breast carcinoma (42). During the past few years, attention has been paid to CTCs as a noninvasive, dynamic, and real-time liquid biopsy to analyze the characteristics of the tumor (43). In this review, we mainly incorporate relevant literature from the last 10 years, with a special focus on the clinical application of CTCs in pancreatic cancer in the current clinical context, where the prognostic value of CTCs in the treatment of pancreatic cancer is the main focus. We also focused on the comparison of the advantages and disadvantages of some imaging techniques and the comparison between CTCs and traditional markers. We briefly outline the classical mechanisms of CTCs generation.

## Epithelial–Mesenchymal Transformation and Circulating Tumor Cells

EMT is the process in which epithelial cells change their polarity, restructure their intercellular junctional complexes, and obtain the phenotypes of interstitial cells under the influence of physiological or pathological stimulus factors. The physiological EMT process begins at the embryonic stage and plays a significant role in the differentiation of organs and tissues during embryonic development. Pathological EMT can adversely cause organ fibrosis and promote carcinoma progression through a variety of mechanisms. The EMT process causes the loss of cell adhesion and changes in the cytoskeleton, which enable tumor cells and the external environment to develop new interactions. The EMT program is highly conservative because the execution program during the embryonic development and carcinogenesis process has high similarity. EMT endows cells with migration and invasion properties, induces stem cell properties, prevents cell apoptosis and senescence, and promotes immune suppression. Through this, it obtains interstitial cell characteristics including infiltration ability and migration ability (44). In various tumor cell lines, scholars have studied multiple signaling pathways that induce EMT, such as the TGF-β signaling pathway, the Notch pathway, and the Wnt signaling pathway. The best-studied signal pathway is TGF-β signaling; research on the combined *in vivo*/*in vitro* carcinogenic model indicates that in the progression of advanced tumors, the activation of the TGF-β pathway can effectively promote the EMT process of cancer cells (45). The activation of these signal transduction pathways will induce the expression of zinc finger transcription factors with high affinity to E-box at the gene transcription level, such as snail, slug, and zeb (46). These transcription factors inhibit the transcription of E-cadherin, causing the loss of adhesion junctions mediated by it. Functioning as a key gatekeeper for cells to maintain an epithelial state, its inhibition is widely involved in various stages of tumor metastasis and progression (46).

**Figure 1:**
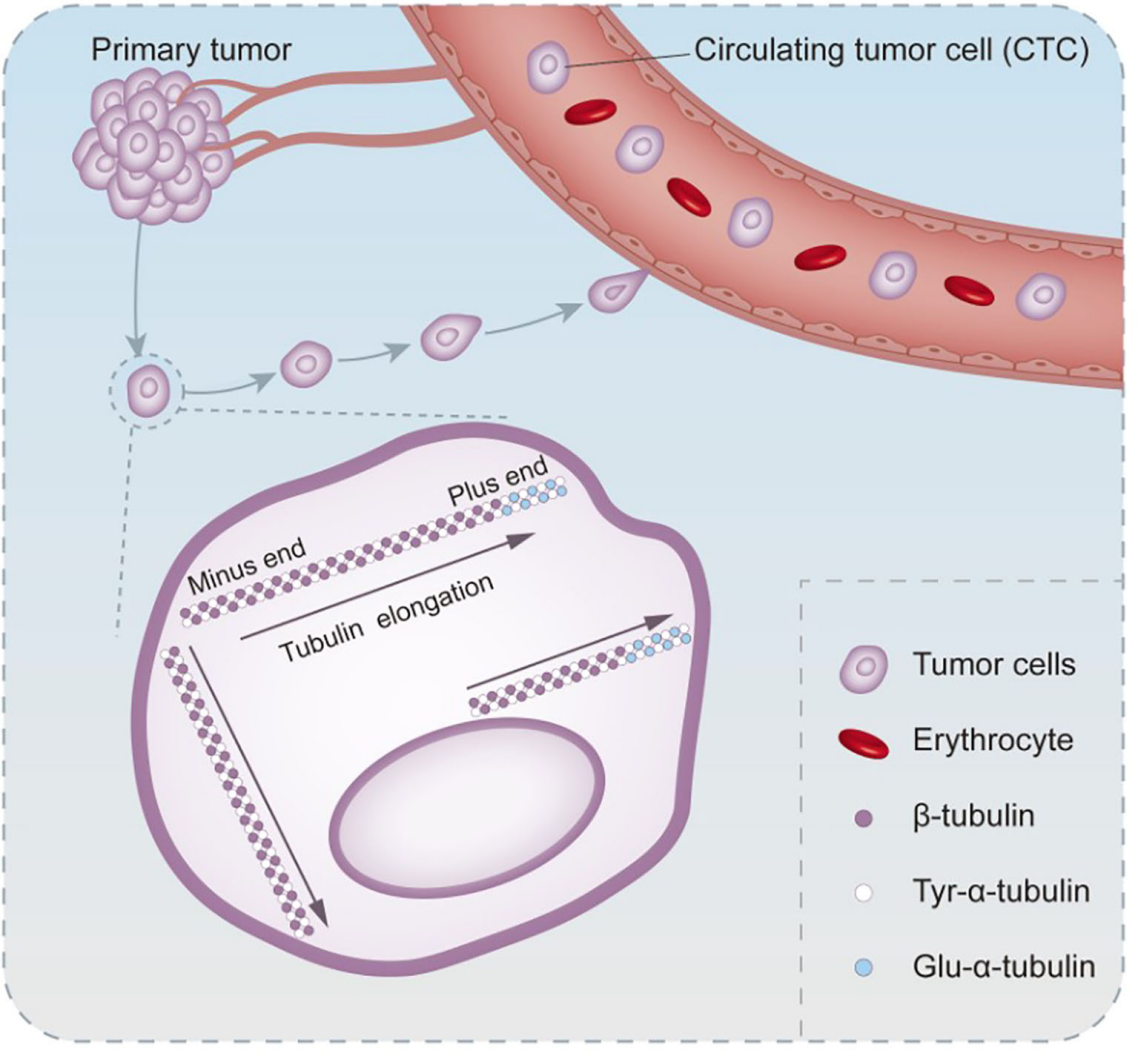
The process of altered microtubule stability in circulating tumor cells undergoing EMT. Post-translational modification of the microtubule constituent protein α-microtubulin occurs when the tyrosine at the carboxyl terminus is replaced by a glutamate residue. Glutamate microtubules are more stable than tyrosine microtubules, and glutamate microtubules extend in the same direction as CTCs migrate.

Changing the cytoskeleton is another important strategy of EMT to promote tumor cell metastasis. According to the cellular tensegrity model theory, cellular morphology is subtly maintained by the tension force of microtubule stents and the counter tension force of actin mesh, which achieve a stable balance to stabilize the shape of cells (47). EMT causes the rearrangement and alteration of the actin cytoskeleton, which is responsible for the dissemination of CTCs (48). In the process of EMT, the stability of microtubules is a key step to improve the efficiency of CTCs transfer. In the post-translational modification of α-tubulin, a constitutive protein of microtubules, glutamic acid residues replaced tyrosine at the carboxyl end. Such alteration is extremely common in human cancers and sarcomas, especially during tumor progression. Tyr-microtubules can only last for 3–5 min, while glu-microtubules last for hours *in vivo* (49, 50). The orientation of glutamate microtubules is the same as that of cell migration, implying that it may be related to the aggressiveness of the tumors (51) (**Figure 1**). Another mechanism that stabilizes microtubules is binding microtubule-associated proteins. Microtubule-associated proteins are a large family with multiple functions. MAP1 and MAP2/tau protein families are the most widely studied in human malignancies. As a soluble microtubule-binding protein, the function of tau is to promote microtubule bundling and stabilization by enhancing the structural strength of microtubules. Compared with bare microtubules, tau-modified microtubules can withstand a greater deformation force in the stress resistance to actin (52). The binding of tau to microtubules and the depolymerization of actin can significantly promote the formation of microtentacles (McTN) (53). This long and dynamic microtubule-rich micro antenna is formed by the extension of the cell membrane of CTCs cells (54). This tau-induced McTN not only increases the reattachment of suspended cells in capillaries to endothelial cells, but also increases the probability of CTCs retention in blood vessels (53). McTN promotes the accumulation of tumor cells of the same type and is of great significance for their survival outside the primary lesion, because effective aggregation can protect CTCs from mechanical damage caused by shear and collision forces and damage from immune monitoring (55).

## CTC Assay for Treatment Management of Pancreatic Carcinoma

It has become a consensus that malignancy is a systemic disease. The therapy of surgically resectable pancreatic cancer is always a combination of local excision and subsequent systemic treatment, and it is therefore helpful in the context of surgical treatment to divide our tumors into localized lesions and systemic lesions. Achieving R0 resection of localized lesions through pancreatic surgery is key to the treatment of localized lesions. For antitumor battles occurring systemically, the detection and characterization of CTCs obtained in the blood can help inform decisions about treatment. For example, patients with a low preoperative CTCs load benefit less from neoadjuvant therapy than those with a high load, and may be better served by surgical treatment alone.

A great advantage of CTCs identification in the treatment process is that it can be dynamically sampled multiple times during the clinical process, so as to distinguish different CTCs subgroups and find specific molecules that drive tumor metastasis and promote chemotherapy resistance. In a study of somatic mutations in complete pathological remission after neoadjuvant therapy (56), the researchers tried to use the genomic analysis of the excised samples combined with liquid biopsy data to reveal the molecular characteristics of the complete pathological remission samples of patients with excised PDAC. Their results showed that after complete pathological remission, even if there are no remaining cancer cells, there are still somatic mutations in the body. This mutation can be monitored after surgery with CTCs and ctDNA. In the tumor self-seeding theory, CTCs colonize the tumor site where it originated through the self-seeding process (57). In animal models, the self-seeding of multiple tumors has been confirmed to be mediated by aggressive CTCs (58). The research of Comen et al. showed that, on the whole, “self-seeding” will change the tumor microenvironment to make it more supportive of tumor growth, including accelerating tumor growth, inducing angiogenesis, and activating matrix recruitment (59). If this microenvironmental change is intervened during the treatment process, theoretically, it should lead to a decrease in CTCs returning to the original site of the tumor. In fact, in locally advanced pancreatic cancer, randomized clinical trials have shown that, compared with chemotherapy alone, adding local radiotherapy to chemotherapy can improve the survival rate of patients (60). Okubo et al. evaluated the change in the number of CTCs before and after treatment in 40 patients with advanced unresectable pancreatic carcinoma. The rate of positive CTCs was significantly higher in patients with progressive disease (45.4%) than with progressive disease or partial response (24.1%), suggesting that the CTCs changes were associated with the tumor response to treatment (61). Bobek et al. (62). proposed a method to isolate CTCs based on cell size and included 24 patients with PDAC. They detected positive CTCs in 66.7% of the patients, and the positive rate of CTCs in patients with T3 PDAC could reach 80%; CTCs can be detected in 77% of patients who have lost the chance of surgery, which provides a hint that patients can be pre-screened before surgery to assess operability. This suggests that the development of new CTCs isolation methods may contribute to more sensitive isolation of CTCs from patient blood. CTCs act as a “bridge” from the primary tumor to the metastasis, and help to find the genotype or phenotype difference between the primary tumor and the metastasis. This difference may be caused by the clonal proliferation process of the primary tumor heterogeneity in metastases and subpopulations in the proliferation of genes or phenotype differentiation (63). If this differentiation results in a difference in sensitivity to chemotherapy drugs, alternative treatment options need to be considered. The current decision-making on the treatment of metastatic tumors is based on the analysis of the primary tumor. In order to reduce the invasive examination of metastatic lesions, “liquid biopsy” of CTCs, which are representative of metastatic lesions, may be a good choice for “experimental biopsy”. If mutations can be found in CTCs using deep sequencing techniques, resistance to therapy can be assessed in real time, which has already been attempted in colorectal and prostate cancers (64, 65). CTCs-assisted personalized therapy has been used in breast and lung cancers. However, susceptible targets have not been successfully identified in pancreatic cancer. We hope that once an effective drug for pancreatic cancer is discovered, based on the information provided by CTCs obtained before and after treatment (such as a patient’s susceptibility genes), it is possible to subclassify patients and select the best treatment for individual cases, such as gene therapy and immunotherapy. In a retrospective study in 2021, Tan et al. studied 155 patients with advanced malignancies receiving anti-PD-1 immunotherapy, including six cases of pancreatic cancer, who were tested for PD1/PDL1 by CTCs obtained from peripheral blood. The results showed that the disease control rate was higher in the PDL1-positive CTCs group (71.56%), and in the remaining patients, the disease control rate was only 39.29%. The reduction in counts and rates of PD-L1-positive CTCs and PD-L1-high CTCs reflected a favorable response to PD-1/PD-L1 inhibitors (66). Haber et al. found T790M resistance mutations in patients with clinical tumor progression who received tyrosine kinase inhibitor treatment by analyzing CTCs (67). *In vitro* models using CTCs help to identify potential therapeutic targets for tumors. For instance, Dimitrov-Markov et al. established a xenograft mouse model using CTCs derived from patients with highly metastatic PDAC. They sequenced RNA from CTCs isolated from patients and identified survivin, a potential therapeutic target for pancreatic cancer. They actually used the CTCs to create a reproducible system to mimic the metastatic process of cancer cells (68). In the future, it is expected that the changes in genotype and phenotype will be continuously monitored during the treatment of pancreatic cancer to ensure timely detection of the emergence of drug resistance, so as to guide changes in treatment strategies. Min Yu et al. used a microfluidic device to efficiently isolate CTCs from endogenous genetically engineered mouse pancreatic cancer models, and through single-molecule RNA sequencing, they found that CTCs are rich in Wnt2 genes (69). The expression of Wnt2 gene in pancreatic cancer cells inhibits cell anoikis, and the non-canonical WNT signaling pathway may contribute to the metastasis of pancreatic cancer. In pancreatic cancer patients, 5 out of 11 pancreatic CTCs (45%) showed abundant WNT signals. The effectiveness of TAK1 in inhibiting this effect establishes a new potential drug target for inhibiting metastasis. Therefore, molecular analysis of CTCs may identify candidate therapeutic targets for preventing the distant spread of cancer. We present some related studies in **Table 2**. In conclusion, the use of CTCs in the clinical management of pancreatic cancer is expected to offer an alternative to “real-time biopsies,” allowing assessment of tumor biological activity. Therefore, detection and characterization of CTCs in pancreatic cancer may be used in preoperative differential diagnosis, prediction of risk of recurrence after surgery, examination of pharmacodynamic biomarkers, detection of treatment resistance profiles, assistance in the designation of treatment strategies, assessment of response to treatment, and assessment of prognostically important. This role is significant, especially in distinguishing pancreatic cancer from neuroendocrine tumors preoperatively and when primary and metastatic tumors are too small to be detected on CT scans.

**Table 2:** Role of CTCs for pancreatic ductal adenocarcinoma (PDAC) treatment.

Author	Year	Cases	CTC positive	Blood source	Treatment	Methodology	Main findings	
Yin, L.	2020	36	100 (100%)	Peripheral blood	Neoadjuvant chemoradiotherapy and curative surgery	ISET, rare cells, and next-generation sequencing	Tumor-related mutations can be detected by CTCs and ctDNA.	(70)
Xu, Y.	2017	40	36 (90%)	Peripheral blood	Chemotherapy (Nab-paclitaxel and gemcitabine)	NE-IFISH	Riploid CTCs could be used to predict the response to the chemotherapy of PC patients.	(71)
Okubo, K.	2017	65	21 (32.3%)	Peripheral blood	Curative surgery, chemoradiotherapy, and chemotherapy	CellSearch system	CTC numbers are a useful tool for predicting therapeutic responses to chemotherapy among patients with advanced pancreatic cancer.	(61)
Tan, Z.	2021	155	127 (82%)	Peripheral blood	Immunotherapy	Pep@MNPs method	The group with PD-L1-positive CTCs had a higher disease control rate (DCR).	(66)
Franses, J.	2020	35	NA	Peripheral blood	NA	CTC-iChip and RNA-sequencing	Pancreatic circulating tumor cell profiling identifies LIN28B as a drug target.	(72)
Wei, T.	2019	100	76 (76%)	Peripheral blood	NA	CytoQuest™ CR system	Significantly reduced CTC counts were observed after chemotherapy in subjects that responded to treatment.	(73)

## The Role of CTCs in Prognosis

The basis for the use of CTCs for early detection of pancreatic tumors is hematogenous dissemination of pancreatic cancer is an early event. As demonstrated in an interesting mouse model of the pancreas, the researchers found that pancreatic cells with characteristics of CTCs circulate in the blood and reach and seed the liver before the primary tumor is found in the pancreas (74). The characterization of CTCs in pancreatic carcinoma not only is an important part of the diagnostic process but also can reveal the risk of tumor recurrence. In addition, mainly due to the subjectivity of tumor plasticity and evaluation criteria, traditional tumor prognostic factors such as tumor stage, pathological grade, lymph node metastasis, vascular and nerve invasion, and tumor resection margin status can better predict the prognosis of patients with pancreatic cancer. However, it can only be evaluated after surgery. CA19-9 can predict the prognosis of pancreatic cancer, but it is susceptible to biliary obstruction, infection, and the patient’s age and gender (75, 76). Conventional prognostic indicators for predicting the prognosis of patients are usually not impeccable. Therefore, there is an urgent need to establish a new and sensitive prognostic method to identify patients with poor prognosis or patients with rapid progress.

Several studies have identified CTCs as a potential minimally invasive mechanism that can be used to analyze patients with primary carcinoma and their risk of subsequent metastasis (77–79). In several different kinds of tumors, CTCs have been identified as a new prognostic biomarker, including lung cancer, locally advanced breast cancer, and liver cancer (80–82). In a large prospective study (*n* = 472 patients), Van Dalum et al. reported that the detection rate is similar before and after surgery (24% to 20%); only the detection of CTCs before surgery is a prognostic factor, while the detection of CTCs after surgery is not associated with a poor prognosis (83). After adjuvant chemotherapy is completed for high-risk colorectal cancer patients, patients with high CTCs have a higher recurrence rate (83, 84). The detection technology used in most studies is FDA-approved CellSearch^®^ technology. A phase 2 study (*n* = 80) in patients with non-metastatic gastric cancer showed that using a technique that claims to have an abnormally high detection rate of CTCs (>90% of patients), CTCs reduction during neoadjuvant chemotherapy is an independent survival prognostic factor (85).

Research on the prognostic role of CTCs in pancreatic cancer is mainly focused on PDAC (86). CTCs have been identified in the blood of patients at all stages of PDAC. Previous studies have shown that there is an association between the presence of CTCs and a poor prognosis (87–91). We list several studies on the prognostic relevance of CTCs and PDAC in the last 5 years (**Table 3**). In the report of Okubo et al., they proved that CTCs are associated with liver metastasis in patients with pancreatic cancer (*p* = 0.0002). They also proved that the presence or absence of CTCs detected by intraoperative molecular RT-PCR detection is associated with a high risk of blood-borne metastasis in patients with pancreatic cancer. Their conclusions indicate that the detection of CTCs can compensate for the insufficient sensitivity and specificity of enhanced CT in detecting disease recurrence (61). However, other studies have shown that the correlation between CTCs and the prognosis of patients with pancreatic cancer is very poor (88, 100). In view of the conflict results of previous studies, Lu Han et al. conducted a meta-analysis of all eligible studies to clarify the prognostic role of CTCs in patients with pancreatic cancer. They included nine cohorts of 268 CTCs-positive patients, and CTCs were significantly associated with poorer PFS (*p* < 0.001) and OS (*p* < 0.001) in patients. Similar results were obtained for subgroup analyses by ethnicity, test method, and treatment method (101). A meta-analysis by Pang et al. included more than 50 patients in nine studies, six of which found a significant relationship between CTCs and OS or PFS (102). A meta-analysis in 2019 included the largest number of studies and patients, including 19 studies with 1,320 confirmed individuals. Their results also support the idea that CTCs-positive patients have worse OS and PFS than CTCs-negative patients, while they suggest that CTCs may serve as a predictive biomarker for pancreatic cancer patients prior to treatment (103). Although there is no consensus on the best detection method and CTCs cutoff value for predicting the clinical outcome of pancreatic cancer, their analysis indicated that CTCs-positive pancreatic cancer patients may have more severe PFS and OS than CTCs-negative patients and the detection of CTCs in peripheral blood may be a promising biomarker for pancreatic cancer detection and prognosis. The differences in the results of previous studies may be due to the small sample size of patients in these studies and the differences in testing and treatment methods.

**Table 3:** Studies evaluating the prognostic value of CTCs in PDAC.

Author	Year	Cases	CTC positive	Blood source	Treatment	Methodology	Main findings	Ref
Gao, Y.	2016	25	100 (100%)	Peripheral blood	Chemotherapy (100%)	SE-iFISH platform	Higher CTC count is a strong indicator for worse OS.	(92)
Okubo, K.	2017	65	21 (32.3%)	Peripheral blood	Curative surgery (14%) Chemoradiotherapy (15%) Chemotherapy (46%)	CellSearch system	The OS of CTC-negative inoperable patients was significantly lower.	(61)
Kulemann, B.	2017	58	39 (67.3%)	Peripheral blood	Curative surgery (64%) Chemotherapy (81%)	CellSearch system	Patients with >3 CTC/ml had a trend for worse median OS than patients with 0.3–3 CTC/ml.	(93)
Ankeny, J.	2018	100	78 (78%)	Peripheral blood	Curative surgery (53%) Chemotherapy (50%)	NanoVelcro CTC assay	CTCs showed promise as a prognostic biomarker for all stages of PDAC.	(86)
Effenberger, K.	2018	69	23 (33%)	Peripheral blood	Curative surgery (32%) Chemotherapy (84%)	MACS Technology	CTCs independently affect the PFS and OS of pancreatic cancer patients	(94)
Bebarova, L.	2018	165	136(82%)	Peripheral blood	Curative surgery (48%) Neoadjuvant chemotherapy (35%)	Epithelial tumor cell assay (ISET; Rare cells)	CTCs are a biomarker for 1-year disease recurrence and mortality	(95)
Zhao, X.-H.	2019	107	84 (79%)	Peripheral blood	Curative surgery (100%)	CanPatrol CTC	Both total CTC number and CTC (EMT) phenotype may act as potential biomarkers for PDAC prognosis.	(96)
Padillo-Ruiz, J.	2021	35	19 (55%)	Central venous and portal blood	Pancreaticoduodenectomy(100%) Chemotherapy (100%)	Isoflux system	The number of free CTCs in portal vein would be beneficial to determine the long-term prognosis before the therapeutic decision.	(97)
Zhang, Y.	2021	31	30 (97%)	Portal blood and peripheral blood	NA	Cyttel Detection kit	High portal vein CTC and mesenchymal CTC numbers were both associated with shorter OS.	(40)
Wang, X.	2021	87	49 (56%)	Peripheral blood	Chemotherapy (100%)	Immunomagnetic microspheres	In patients with advanced pancreatic cancer treated with chemotherapy, CTC positivity was associated with shorter PFS.	(98)
White, M.	2021	34	31 (91%)	Portal blood and peripheral blood	Curative surgery (100%)	CellSearch system	Portal blood CTC count but not peripheral blood CTC counts were significantly correlated with OS.	(99)

However, the prognostic role of CTCs in pancreatic cancer is not without its limitations. In a previous study, in 2,183 blood samples from 964 metastatic carcinoma patients, 36% (781 of 2,183) of the specimens had only 2 CTCs (104). The rarity of carbon tetrachloride in the peripheral blood of patients with non-metastatic cancer greatly limits its use as a predictor of metastasis. The reason may be that the diameter of CTCs is about 25 mm, which is too large for them to pass through capillaries (about 8 mm in diameter) (104). Studies in animal models have shown that most of the radiolabeled tumor cells injected into blood vessels are trapped in the capillary bed of the first organ that cancer cells reach (similar to the liver’s first-pass elimination effect), which is difficult to detect in peripheral blood (105). However, this problem is not insoluble. Rahbari et al. studied the difference in the interval distribution of CTCs in patients with colorectal cancer, and the results show that the detection rate of CTCs is higher in tumor drainage (mesenteric) vessels compared with peripheral venous blood. Theoretically, the concentration of CTCs detected in the portal blood of patients with pancreatic cancer is higher than that of peripheral blood. Therefore, in patients with pancreatic cancer, the count of CTCs in portal blood is expected to be an early indicator of liver micrometastasis. In a cohort study(*n* = 29) (106), the researchers used ultrasound-guided transhepatic puncture to analyze the CTCs in the portal blood of patients with advanced pancreatic cancer. CTCs were detected in 29 cases of portal vein blood, and the absolute number of pancreatic cancer cells circulating in the portal vein was significantly higher than that in peripheral blood. The study found that the portal vein CTCs count is highly correlated with intrahepatic metastasis. The OS of patients with portal vein CTCs exceeding 150/7.5 ml is significantly shortened, suggesting that patients with advanced pancreatic cancer have a poor prognosis. The results show that the analysis of portal vein CTCs is a powerful method for the prognosis of patients with advanced pancreatic cancer. It is worth noting that due to the sensitivity difference between different technologies, the CTCs cutoff shown by one CTCs detection technique is a prognostic factor, and another technique cannot be inferred. The timing of CTCs detection is also critical. Positive CTCs before, during, or after surgery may have different biological and clinical significance. In many studies, portal blood was obtained from patients undergoing radical pancreatic surgery for CTCs, and it was clear that the detection rate was higher in portal blood than in peripheral veins; however, it is difficult to determine whether this difference is related to the compression of the cancerous tissue caused by the surgery or to the elimination effect of the liver. If future studies can obtain portal blood, central venous blood, and peripheral blood simultaneously and compare the levels of CTCs in the three types of blood, and correlate them with tumor recurrence and time of liver metastasis, this mystery will be revealed.

The ultimate fate of CTCs depends on their resistance to denervation, immune killing, and apoptosis. Some studies have explored the relationship between the apoptosis level of CTCs and the prognosis of breast cancer. They found that the higher the expression level of the apoptotic factor BCL-2 in CTCs, the better the prognosis of patients. This may be the result of a multifactorial effect, in addition to the possible activation of immune killing by more CTCs and the association of less deformable CTCs with vascular anatomy, and tumor metastasis should also be considered (107).

## Summary

In the era of multidisciplinary collaborative diagnosis and treatment, pancreatic cancer is still one of the most malignant tumors. As a promising direction of tumor research, CTCs have actively explored the treatment of pancreatic cancer and its prognosis. With the continuous development of various CTCs enrichment and detection equipment, it can be speculated that there will be more surprising discoveries in the future to challenge the existing practice of diagnosis and treatment of pancreatic malignancies. As new assay technologies continue to emerge and the heterogeneity of CTCs becomes better understood, we need in-depth genetic and gene expression pattern studies of different types of CTCs, and we may need CTCs assays that are specific to different types of tumors, rather than one assay technology that covers all cancer types. In fact, a tumor cell-specific assay should precisely match the characteristics of that tumor, in line with the concept of precision medicine, and one of the disadvantages of CA199 compared to CTCs is that it cannot be specific to each tumor. In the future, we believe that “whole process monitoring” of CTCs will provide more information for decision-making in the treatment of tumors, such as preoperative differential diagnosis, prognostic assessment, real-time monitoring of drug resistance mutations during treatment, assessment of treatment response, and timely detection of micrometastases. All of these aspects are of most interest to clinicians battling pancreatic cancer. Although we have repeatedly discussed the increased risk of metastasis and spread of pancreatic cancer with invasive testing compared to “liquid biopsy”, more prospective studies are needed to prove this point in the future.

Similarly, do diagnostic and therapeutic interventions (e.g., surgery and chemotherapy), and access to CTCs in portal blood or even in pancreatic ducts, result in more CTCs being released into the bloodstream and, if so, does this release alter the prognosis of pancreatic cancer? It is conceivable, given the right conditions, whether test specimens obtained from different routes would alter the results; which test specimens obtained from different routes would be most appropriate for surgical and non-surgical patients, respectively; and whether portal blood or even pancreatic fluid would be preferable to central venous blood and peripheral blood when considering convenience and economic cost. New and surprising findings may challenge our current paradigm and medical practice. Admittedly, with only one assay, the CellSearch system, now in the transitional phase of FDA approval to clinical application, new assays must be validated in clinical trials to prove their efficacy. In any case, it is always helpful to explore all aspects of the utility of CTCs as one of the few new markers now in development. It is hoped that this review will provide some insight into the clinical application of CTCs and the clinical validation of CTCs as new biomarkers.

## Author Contributions

KL did the bibliographic search. KL, XW, and XZ wrote the initial manuscript. ZL, SH, and RL critically revised the manuscript and actively contributed to it. All authors contributed to the article and approved the submitted version.

## Funding

RL is supported by the 2022 Henan Province Key R&D and Promotion Special Support Project.

## Conflict of Interest

The authors declare that the research was conducted in the absence of any commercial or financial relationships that could be construed as a potential conflict of interest.

## Publisher’s Note

All claims expressed in this article are solely those of the authors and do not necessarily represent those of their affiliated organizations, or those of the publisher, the editors and the reviewers. Any product that may be evaluated in this article, or claim that may be made by its manufacturer, is not guaranteed or endorsed by the publisher.
